# Emotional Responses Toward a New Research Policy Among Academics in a Chinese University

**DOI:** 10.3389/fpsyg.2021.777472

**Published:** 2021-11-23

**Authors:** Hua Lu, Xiaorong Zhang

**Affiliations:** ^1^School of Foreign Studies, Anhui Polytechnic University, Wuhu, China; ^2^School of Foreign Studies, Anhui Normal University, Wuhu, China

**Keywords:** emotion, emotion regulation, research practice, research policy, university EFL teacher-researchers

## Abstract

Teacher emotion has received prominent attention in the field of education as they are closely related to teacher identity and teachers’ well-being. While many previous studies have taken teachers’ emotions in teaching as their research focuses, this study investigated university English as a foreign language (EFL) teacher-researchers’ emotions and emotion regulation strategies in research in the context of a new research policy. One-on-one semi-structured interviews were conducted with seven EFL teacher-researchers at a Chinese university which implemented China’s new research policy of breaking the “five-only,” supplemented by the analysis of narrative frames and the institutional research documents. The data revealed that university EFL teacher-researchers experienced wide-ranging and diverse emotions at the micro-level, meso-level, and macro-level of research, with different attitudes toward the new research policy. They also employed multiple strategies of antecedent-focused and response-focused approaches to regulate emotions in research. This study helps unpack the complexity of emotions experienced by university teachers in research, and also calls for the attention of stakeholders to pay to the emotions and well-being of university EFL teachers.

## Introduction

There has been an increasing number of studies on university EFL teachers’ research practice and engagement in the publish-or-perish culture (Allison and Carey, 2007; Borg, 2007, 2009, 2010; Barkhuizen, 2009; Borg and Liu, 2013; Long and Huang, 2017; Teng, 2019). In China, most universities have been adopting the practice of taking research productivity as the main criteria in recruitment, promotion system, and performance evaluation (Wang and Han, 2011). Under such circumstances, many university EFL teachers have complex and mixed emotions toward research, such as enthusiasm, frustration, stress, and anxiety (Gu et al., 2014; Lee, 2014). Understanding teachers’ emotions in research practice is important in the sense that their emotions strongly affect their research engagement, research productivity, and researcher identity construction (Day and Harris, 2016).

In China, the “five-only” evaluation system which complies with the publish-or-perish culture has long been practiced in many higher education institutions. The “five-only” refers to “value manuscripts only,” “value academic credentials only,” “value professional titles only,” “value awards only,” and “value academic titles only” (Luo, 2020). Nevertheless, the Chinese government has released a new research policy stating its position on breaking the “five-only” after realizing its detriment of fostering a utilitarian academic environment in recent years (Cao, 2019). This shift is bound to pose some challenges and possible emotional flux for Chinese university EFL teachers since their research engagement is greatly influenced by the socio-institutional context (Liu and Borg, 2014; Zhou and Zhang, 2016).

While a number of scholars have studied teacher emotions in different contexts such as in English language teaching (Benesch, 2013), in second language teaching (Barcelos and Ruohotie-Lyhty, 2018) and in educational reforms (Zembylas, 2010), teachers’ emotional experiences in research practice remain understudied. The present study seeks to explore what emotions Chinese university EFL teachers experience in research, whether and how they regulate their emotion in the context of the new research policy. Specifically, the study aims to explore the following two questions:

• What are the emotional experience of the EFL teacher-researchers in responding to the research policy changes?
• How do EFL teacher-researchers navigate the complex emotional experiences in responding to the research policy changes?

## Literature Review

### Teacher Emotion and Emotion Regulation

Teacher emotion has received increasing attention in the last few decades. Scholars have found that teacher emotion plays an important role in teachers’ personal and professional development as it is closely related to teachers’ cognition and learning, teachers’ teaching behaviors and teaching interaction (Hargreaves, 2000; Golombek and Johnson, 2004; Zembylas, 2007; Day and Qing, 2009; Deonna and Teroni, 2012). Researchers have commonly classified teacher emotions into a dichotomy of positive and negative types (Benesch, 2013). Positive emotions refer to the positive subjective experience and pleasant emotions such as joy, happiness, hope, pride, love, and excitement that a teacher produces when approaching a certain goal. Correspondingly, negative emotions are the negative subjective experience and unpleasant emotions such as frustration, disappointment, anxiety, anger, fear, and sadness that occur when a teacher is far away from his or her goals (Day and Lee, 2011). Besides this classification, there are some other types of teacher emotions. For example, Lazarus (1991) divided teacher emotions into positive, negative, fringe, and non-emotions, whereas Lee and Yin (2011) put teacher emotions into categories of positive, negative and mixed ones. In spite of the different classifications, scholars unanimously hold the view that teacher emotions are complex and multifaceted (Gu, 2016; Han and Xu, 2021). Given the complex nature of teacher emotions, researchers have unsurprisingly identified teachers’ rich emotions in teaching and research, such as joy and satisfaction (Hargreaves, 2000), enjoyment and contentment (Uzuntiryaki-Kondakci et al., 2021), enthusiasm (Kunter et al., 2011), pleasure and pride (Sutton and Wheatley, 2003), anger and frustration (Chang, 2013), stress (Becker et al., 2014), sadness and dissatisfaction (Hagenauer and Volet, 2014), inadequacy and confusion (Uzuntiryaki-Kondakci et al., 2021).

In addition, scholars have also paid special attention to teacher emotion regulation. Researchers have found that emotion regulation is not only conducive to enhancing teachers’ teaching effects (Sutton, 2004), but also closely related to teachers’ well-being (Yin, 2016). According to Eisenberg and Fabes (1992), emotion regulation refers to “the process of initiating, maintaining, modulating, or changing the occurrence, intensity, or duration of internal feeling states and emotion-related physiological processes” with the intention of achieving one’s goals (p. 137). It has been pointed out that researchers are at the initial stage of studying teachers’ emotion regulation (Sutton and Harper, 2009), however, scholars have already identified two broad regulatory approaches with several strategies, respectively: the antecedent-focused emotion regulation approach and the response-focused approach. According to Gross and John (2003), antecedent-focused regulation occurs before an emotion has fully bloomed while response modulation occurs once an emotion is already generated. In Gross (1998, 2015) emotion regulation framework, antecedent-focused emotion regulation consists of situation selection, situation modification, attention deployment, and cognitive change, whereas response-focused emotion regulation involves the emotional response modulation of the physiological, experiential, or behavioral aspects. Further, Gu and Gu (2019), and Han and Xu (2021) have identified five and three regulation strategies of response-focused approach, respectively, based on their empirical study on university teachers’ emotion regulation strategies. The two approaches and specific strategies of emotion regulation adapted from Gross (1998, 2015), Gu and Gu (2019), and Han and Xu (2021) are shown in Table 1.

**TABLE 1:** Emotion regulation approaches and strategies adapted from Gross (1998), Gross (2015), Gu and Gu (2019), and Han and Xu (2021).

**Emotion regulation approaches**	**Emotion regulation strategies**	**Definition**
Antecedent-focused emotion regulation	Situation selection	Deliberate approaching or avoidance to some places or situations which might trigger certain emotions
	Situation modification	Tailoring a situation to modify its emotional impact
	Attention deployment	Selecting which aspect of a situation to focus on
	Cognitive change	Choosing which meaning out of the many possible ones to attach to a situation
Response-focused emotion regulation	Adapting	Adjusting one’s mentality to accommodate the environment or to accept requirements
	Taking actions	Actively taking certain actions to enhance positive emotions
	Communicating	Communicating with other people to decrease negative emotions
	Suppression	Controlling oneself from showing and/or experiencing certain emotions
	Relaxation	Using relaxation techniques to change one’s emotional response to a situation

Besides the common regulation goals of increasing positive emotions and decreasing negative emotions (Gross, 1999) in themselves, teachers have also been reported to use emotion regulation strategies to achieve other goals, such as the instructional goal of effective teaching, the purpose of nurturing good teacher-student relationships, the aim of maintaining ideal and professional teacher images, the purpose of easing burn-out, and the goal of improving job satisfaction (Sutton, 2004; Hosotani and Imai-Matsumura, 2011; Lavy and Eshet, 2018; Taxer and Gross, 2018). However, with the continuous development of studies on teacher emotion, researchers have gradually realized that the relation between emotion regulation and cultural differences cannot be ignored (Mortenson, 2006; Day and Lee, 2011). For example, Sutton and Harper (2009) found that most American teachers in their study used response modulation to increase their positive emotions such as happiness, while Gong et al. (2013) reached a conclusion in their study on Chinese teachers’ emotion regulation goals that more Chinese teachers used response modulation to down-regulate their negative emotions such as anger and frustration rather than expressing positive emotions.

Scholars have made prominent research findings in teacher emotion in the past decades, however, they have also noticed that Western societies have made dominant contributions in this field (Uitto et al., 2015). Given that social-cultural context plays a crucial role in the accepting or rejecting of ideas and practices in different parts of the globe (Dimmock and Walker, 2000), and research findings from Western societies have been proven to be unsuitable for Eastern countries (Qian et al., 2017; Chen, 2019), exploring teachers’ emotional experiences and regulation strategies in Asian countries such as China is in timely and urgent need. In recent years, some scholars have explored teachers’ emotions in the Chinese context such as Chinese teachers’ emotion regulation goals and strategies in teaching (Gong et al., 2013; Yin, 2016) and Chinese university teachers’ emotion regulation strategies in research (Gu, 2016; Gu and Gu, 2019). Both studies found that Chinese teachers used various response-focused and antecedent-focused strategies to control their emotions. However, empirical research on Chinese university EFL teachers’ emotions in research practice in the context of China’s new research policy has rarely been found. Thus, it is still of necessity to explore this matter further in the current Chinese sociocultural context with the release of this new research policy.

### Breaking the “Five-Only”

In China, the “five-only” evaluation system which values manuscripts published in highly prestigious journals, research grants, and awards from the central and provincial governments, teachers’ academic credentials and professors’ academic titles has long been practiced in higher education institutions (Bie and Yi, 2014). Under its influence, most universities in China display a research-oriented institutional culture which places an overemphasis on teachers’ research productivity in promotion systems (Borg and Liu, 2013). As a result, university teachers in China, regardless of their age and professional title, are overwhelmed by the task of writing and publishing manuscripts (Fu et al., 2019). In addition, the academic pursuit of university teachers has been largely constrained by this evaluation system because many teachers have to adjust their research focus to cater to the interest of some journals if they want to get manuscripts published. This utilitarian practice has been criticized by scholars and experts in academia (Fan, 2019).

Having realized the harms of the “five-only” evaluation system, the Chinese government has released a number of documents stating its position on breaking the “five-only” (Cao, 2019). However, some scholars have expressed their concerns about the repercussions of this new policy. They point out that, as a complex product of long-term administrative centralism, managerialism and utilitarianism, “five-only” is not formed overnight and it cannot be abolished completely in short term (Wang, 2021). Furthermore, there is a lot at stake when it comes to establishing a new evaluation system in higher education, such as what this new system should be, to what extent it can guarantee fairness in evaluation, and how to establish such a fair evaluation system (Lu, 2019).

Under circumstances like this, it is greatly complex and challenging for Chinese university EFL teachers to conduct research and construct their researcher identity. Compared with teachers in other disciplines, EFL teachers are more susceptible to the influence of sociocultural context (Borg and Liu, 2013). Since China implemented the policy of reform and opening up in the 1970s, there has been an upsurge in English learning across the country (Gao et al., 2011). For college students, obtaining a certain level of English certificate such as the College English Test Band 4 and Band 6 has become one of the necessary conditions for graduation and one of the prerequisites for obtaining a good job. With the drastic increase in the number of English learners in the country, the demand for English teachers has correspondingly increased (Li, 2020). In the past few decades, English majors who have obtained a master’s degree are considered to be qualified to teach college English, and their academic research ability is basically not taken into account in the process of recruitment (Liu and Borg, 2014). Compared with teachers with doctorates in other disciplines, many EFL teachers in China lack systematic academic training and solid academic accumulation (Ellis et al., 2002). Although some English majors with doctorate degrees have joined the team of EFL teachers in recent years, they account for only a small proportion of the total (Liu and Borg, 2014).

As compared to faculty members of other subject areas, EFL teachers have a weak tradition in research with inadequate research competence (Dai, 2009). Burdened with a heavy teaching workload of ten to twenty periods of classes per week, many university EFL teachers are unable to commit sufficient time and energy to research practice (Hong, 2014; Xu, 2020). Even when they do, they tend to mainly focus on the introduction and review of the academic achievements of English-speaking countries (Gao et al., 2000). Furthermore, there are relatively few foreign language academic journals in China, which also stifles EFL teachers’ enthusiasm for research since their publishing needs are difficult to meet (Wang and Han, 2011). The discouraging factors also include lack of institutional and financial research support, lack of research teams, and lack of experienced team leaders (Teng, 2019). Therefore, EFL teachers’ research engagement and the quality and quantity of their research outcomes in terms of original research are unsatisfactory (Dai, 2009). Luckily, the issue of research competence has caught the attention of EFL teachers themselves and other stakeholders in higher education since they have realized that research engagement can help teachers’ identity formation and professional growth (e.g., Xu, 2016; Yuan, 2017; Rahimi and Weisi, 2018; Qian and Huang, 2019; Rahimi et al., 2019).

With the implementation of this new research policy of breaking the “five-only,” it is worthwhile to explore the specific emotions and the emotional changes, if any, university EFL teacher-researchers experience in research practice. Since teacher emotions reflect teachers’ self-cognition and influence their identity construction, decision-making, and behaviors (Hargreaves, 1998; Schutz and Zembylas, 2009), teachers’ emotions in research inevitably reflect teachers’ research beliefs and affect their research performance and professional development. The present study aims to gain an in-depth understanding of Chinese university EFL teacher-researchers’ emotions in research through exploring the specific emotions they experience in the context of China’s new research policy of breaking the “five-only” and what strategies they use to regulate their emotions in this context.

## Methodology

### A Narrative Study

Narrative inquiry was adopted as a research method in this study. As a common research approach in social sciences (Atkinson, 1998), narrative inquiry studies people’s experiences as stories and how people make meaning of their experiences by telling and retelling their stories (Clandinin and Connelly, 2000). Connelly and Clandinin (2006) pointed out that narrative inquiry was a powerful research method in the field of education due to its appeal of representing and understanding life experiences as stories. In the past several decades, there has been a notable academic interest in the interaction between narratives and people’s personal experience in the background of different social, cultural, and political contexts (Clandinin and Connelly, 1996).

Since narrative inquiry is helpful in collecting data on participants’ storied experiences, attitudes, and opinions about their experiences (Barkhuizen and Wette, 2008), it was used to collect qualitative data in the present study. This study used narrative frames and semi-structured interviews to collect data on participants’ basic background information, research experiences, feelings, and emotions in research practice, attitudes, and views of research and research policy.

### Research Context and Participants

The present study was conducted in a typical public university in the south-central part of China. Public universities like this account for roughly 94% of all higher education institutions in China (Wang, 2018). Even though these universities do not belong to the Projects 211 and 985 launched by the Chinese government with the purpose of promoting the research output of elite universities, they have been inevitably influenced by the competitive state of higher education development in the country, and this university is no exception. Under the influence of the publish-or-perish academic culture and the desire to be a nationwide higher-ranking university, it gives a high priority to research by taking a number of measures. For example, it has adopted a periodical evaluation system with 3 years as an assessment cycle for teachers’ research productivity in addition to its consistent research-oriented teacher promotion system. In such an institutional culture, teachers have been constantly encouraged to apply for research grants funded by the provincial government or above and to get manuscripts published in prestigious journals. Considering factors of feasibility, familiarity, accessibility as well as time and costs (Hatch, 2002), this public university was selected as the research site of this study.

There are 83 EFL teachers in this university, however, only approximately 24% of them have research productivity according to the official statistics provided by the English department in its 2020 annual work report. Considering that not many EFL teachers at the research site are actively engaged in research, the authors used purposive sampling to select participants. In this way, selected cases can make the most out of limited resources (Patton, 2002), provide rich information, and achieve maximum variation on different aspects such as professional title, personal and educational backgrounds (Huberman, 1989). Participants were chosen based on the following criteria. The first one is that the selected participants have dual roles of teacher and researcher. They are both engaged in EFL teaching and research practice. The other one is that they have already had certain research achievements such as having published one academic book or having published manuscripts in the university’s evaluation cycle of 3 years. Based on the criteria and the principle of maximum variation, nine EFL teacher-researchers were approached, and seven of them agreed to participate in this study. Participants Edward, Alice, Zoe, Peter, Betty, Mark, and Serena (pseudonyms) vary in gender, years of teaching, personal and educational backgrounds, professional title, and teaching subject (see Table 2).

**TABLE 2:** Background information of the participants.

**Name**	**Gender**	**Years of teaching**	**Professional title**	**Education qualifications**	**Teaching subject**
Edward	Male	18	Professor	Ph.D. in Foreign Linguistics and Applied Linguistics MA in English Linguistics and Literature BA in English language education	English linguistics
Alice	Female	20	Associate Professor	MA in British literature BA in English language education	British literature
Zoe	Female	17	Associate Professor	MA in English teaching pedagogy BA in English language	College English
Peter	Male	15	Associate Professor	MA in translation BA in English language	English-Chinese translation
Betty	Female	21	Lecturer	MA in American literature BA in English language education	American literature
Mark	Male	8	Lecturer	MA in second language acquisition BA in English language	English writing
Serena	Female	4	Lecturer	MA in intercultural communication BA in English language	Cultures of English-speaking countries

### Data Collection

Data of this qualitative study was collected and triangulated through three methods, namely, narrative frame, semi-structured interview, and document analysis. Since narrative frame serves as a research instrument to collect data from participants’ storied experiences (Barkhuizen and Wette, 2008; Barkhuizen, 2011), this study used it to gather basic information of participants and to help them reflect upon their practices. The narrative frame provided to participants covers several themes, ranging from basic information about participants’ educational background and teaching experience to their attitudes toward research. Semi-structured interviews were conducted by the first author in this study. As a quintessential research method in qualitative studies, interview is highly efficient in collecting data (Kirkevold and Bergland, 2007). All the interviews were conducted face-to-face and one-on-one. Each interview was between 40 and 60 mins. In the interviews, participants were asked to describe their emotions unfolding in research practices, to review their research experiences, and to talk over their attitudes and feelings toward research policies. The key interview questions are presented in the “Appendix.” All the interviews were conducted in Chinese and audio-taped, and then transcribed verbatim and translated into English. After that, transcripts and translation of the interviews were sent back to the participants for verification. In addition, document analysis was also employed in this study to collect data since this method can access ready-made data sources and provide additional detailed information about research participants’ contexts and settings (Bowen, 2009). The university has posted online the documents related to the changes in the institutional research policy after the government’s release of breaking the “five-only” policy. This study collected the pertinent official documents from the school’s website with the permission of the dean of the School of Foreign Studies.

### Data Analysis

Data analysis occurred along with the process of data collection. The collected data were analyzed by means of thematic analysis (Braun and Clarke, 2006), content analysis (Fraenkel et al., 2012), and the constant comparative method (Corbin and Strauss, 1990). This study examined university EFL teacher-researchers’ emotions through the framework of the multifaceted nature of education and teacher development (Douglas Fir Group, 2016). The authors followed the six steps of thematic analysis to analyse data collected through interviews (Braun and Clarke, 2012; Clarke and Braun, 2013). Firstly, we repeatedly read the interview transcripts to be familiarized with data. Then, we carefully generated initial codes of teachers’ emotions, such as “be under a lot of pressure,” “be very happy,” “feel frustrated,” based on extracts related to participants’ emotions and feelings of research. After that, the initial codes were revised in consulting literature. For example, the initial codes “too much pressure,” “be under pressure,” “external pressure” were subsumed to “stress,” which was one of the negative emotions reported in studies on teacher’s emotions in research (Lee, 2014; Gu, 2016). Then we searched for themes that could gather all relevant data. In this study, themes related to the teachers’ emotions at the micro-level of research itself, the meso-level of institutional system, and the macro-level of national policy emerged. The three themes were reviewed and named as “emotions generated by intrinsic value of research at micro-level,” “emotions generated by institutional system at meso-level,” and “emotions generated by social expectation at macro-level” (see Table 3). Content analysis was used to analyse institutional research policy documents and participants’ narrative frames so as to triangulate participants’ emotions and attitudes toward research and research policy. Also, we compared emotions and regulation strategies of participants of the same professional titles and those of different ones by using the constant comparative method. As for emotion regulation strategies, two approaches were revealed through the process model of emotion regulation: antecedent-focused regulation and response-focused regulation (Gross, 1998, 2015; Gu and Gu, 2019). The former included strategies of situation selection, situation modification, attention deployment and cognitive change, and the latter consisted of suppression, adapting, taking actions, and self-improving (see Table 4).

**TABLE 3:** The participants’ emotions in research.

**Themes**	**Emotions**	**Participants (*n*)**	**Extracts (*n*)**
Emotions generated by	Enjoyment	5	6
intrinsic value of research	Pride	3	8
at micro level	Happiness	3	4
	Stress	6	16
	Frustration	6	15
	Inadequacy	5	6
	Pain	3	5
	Anxiety	3	4
	Doubt	2	6
	Mixed feelings	2	3
Emotions generated by	Satisfaction	3	6
institutional system at	Powerlessness	3	5
meso level	Dissatisfaction	3	4
Emotions generated by	Confusion	3	4
national policy at macro	Indifference	3	3
level	Approval	2	2

**TABLE 4:** The participants’ emotion regulation approaches and strategies.

**Approaches**	**Strategies**	**Participants (*n*)**	**Extracts (*n*)**	**Example from the responses**
Antecedent-focused	Situation selection	1	4	“I don’t think too much about that in my life”
	Situation modification	1	1	“You can’t just keep your head down and do research in your own way”
	Attention deployment	2	4	“I usually think about some of my success in the past, or think about other things”
	Cognitive change	2	2	“You have to convert pressure into motivation, and you should think about the positive side”
Response-focused	Suppression	2	2	“We teachers feel very much at a loss, but there is nothing we can do about it”
	Adapting	3	3	“Because in the promotion system, it clearly lists which manuscripts are high-level manuscripts and which level of research grants are required, these naturally become the main focus of my research work”
	Taking actions	5	5	“I got up early in the morning and then spent about one and a half hours reading and writing manuscripts”
	Self-improving	6	6	“I will further polish my application and continue to apply for grant next time”

In order to ensure the reliability and trustworthiness of the study, the first author and the second author analyzed the data independently. The inter-coder agreement was 86%, and we worked on the rest with discussion and consulting literature. Also, the results of the data analysis as well as the raw data were presented to the participants for accuracy checking.

## Findings

The present study identified a range of emotions that participants experienced at the micro-level of research itself, at the meso-level of institutional system, and at the macro-level of national policy. This section will illustrate participants’ different and varied emotions at each level and their emotion regulation strategies.

### University English as a Foreign Language Teacher-Researchers’ Emotions in Research

Data analysis points to positive and negative emotional experiences faced by the EFL teachers. Positive emotional experiences were contributed by sense of recognition and personal learning. In sense of recognition, participants associated the new research policy as a way for them to get their achievements in publications and securing grants recognized. For example, Edward believed that this new research policy is fair as he recalled his past experience of the arduous process of publishing and getting a research project supported by the National Social Science Foundation in 2011. It was the biggest academic achievement in terms of getting research grants for him. The success has naturally made him very proud of his hard work and led to his fruitful research productivity such as publishing books and publishing manuscripts in top-tier journals in the following years.

“I was very happy of course, because that means my continuous efforts were recognized by the academia, and it was also an important success on my own research road. The support of this national research grant is also conducive to my further research. So, getting it is really a happy thing.”

Thus, securing a prestigious grant and publishing in top-tier journals as “very important indicators” to his researcher identity. In a similar vein, Serena stated in her narrative frame that she could see her future self getting more and more involved in research as a way to contribute toward improving the quality of education. In the semi-structured interviews, she reiterated that the satisfaction felt when she was able to secure grants and publish made all the hard work worthwhile:

“I think the purpose of research is to get it published, right? But the research process is definitely hard, especially for us liberal arts majors. Getting research grants and publishing manuscripts are very difficult for us, but it is really happy if we get them.”

The participants seemed to associate positive emotional experiences with two major tasks which are securing grants and publishing. Participants benchmarked a sense of recognition with success in these two tasks. Zoe seemed to be the only participant who associated positive emotional experience as a researcher with the new research policy:

“I think this is of course a good thing. We’re no longer fighting ruthlessly for publishing manuscripts or getting research grants like before, right? I think I no longer feel very eager for quick success like publishing a certain number of articles. Maybe now I can sit down and do it slowly according to my own research interests since there is no quantitative requirement now.”

As a researcher, she found excitement in her research interests as she shared that “I can still learn new things and do different things, which make me feel quite fulfilling.”

Surprisingly, in the narrative frames, most participants wrote that they did not do research as frequently as expected, since they were mainly doing it to meet the requirements of their university. Furthermore, this study found that those who have been largely driven to do research by external powers doubted the necessity of research. For example, Betty used the metaphor of “disjoint parallel lines” to describe the relationship between teaching and research, because she believed that “there was basically nothing overlapping between teaching and research.” Similarly, in Mark’s opinion, it was meaningless to do research:

“There is no point in research, it is for quick success and instant benefits. It is of little use to teaching. Doing it is just for the purpose of passing the KPI assessment. I personally feel that there is no need for college English teachers to do too much research, because we mainly teach students’ language, right?”

The negative emotional experiences can be attributed to the uncertainties toward the new research policy, past failures in research, heavy workload, and gaps in research skills. The lack of understanding on the new research policy had generated negative emotions among the participants. Participants had been in the Chinese academic for 4–21 years. They knew extensively the previous policy and had been charting their career based on the previous policy. The introduction of the new policy had caused confusion on measurement of academic performance as well as the repercussion of not meeting the desired target as stipulated in the new policy. Both in the narrative frame and semi-structured interview, Mark lamented that:

“They think that publishing manuscripts should be given a 3-year KPI assessment. This is a corporate management thinking mode. It shouldn’t be like this in research, all right? I think the whole system is problematic.”

Likewise, Serena expressed the similar confusion regarding the changes brought about by this new research policy:

“Also, now the policy of breaking the “five-only,” but what will happen later then? How to establish a new evaluation standard? We are not sure about the new standard. We don’t know about that.”

In addition, Betty also showed her dissatisfaction by complaining that the changes in institutional policies had a negative impact on their research, because now they were overwhelmed with “more things to do,” besides trying to meet “the difficult demands for high-quality manuscripts,” they also needed to “taking part in teaching competitions,” and “guiding students to take part in various competitions” as a result of the changes in institutional research policies. A review of the university performance indicator document revealed that research output is no longer regarded as the only thing valued in promotion and 3-year assessment, teachers’ teaching competition awards and tutoring students are given more priority than before. As a result, teachers need to be engaged in these two in addition to research practice.

The negative emotional experiences felt by participants could be attributed to the past failures in research, the heavy workload in teaching, administration, and inadequacies in doing research. For example, Alice honestly admitted that what she remembered the most in her research life were the many setbacks she had in years. In her narrative frame, she mentioned one of her past experiences of a manuscript publication failure. After submitting a manuscript to a prestigious journal, she was rejected in a few months because the reviewers said that her language was unsuitably westernized. She later commented that this was one of the most impressive moments in research because “being denied by others” made her feel frustrated, and that feeling lingered on. Similarly, Edward, Zoe, and Mark all expressed their frustration at being rejected for manuscript publication or grant applications. When Edward reflected on his journey of becoming a researcher, he remarked that:

“Sometimes my manuscript cannot be published or is rejected, and my grant application is not approved, then I feel very frustrated and depressed. This is human nature because my own efforts are not recognized.”

Participants also felt that they had to reduce the time allocated for research-related activities because of their heavy teaching workloads and administrative duties. In the narrative frames, participants reiterate their commitment to teaching, such as “Teacher is my main identity,” “Teaching is my top priority.” Naturally, heavy teaching workloads such as ten to twenty periods of classes per week take up much of their time and energy. In a semi-structured interview, Alice commented that “my team has been busy participating in teaching competitions for the past 2 years, so my research has been held up.” A review of the English department’s teaching arrangement documents substantiates the participants’ claim that teaching takes up most of their time and academics are expected to take part in teaching competition. As such, to evenly allocate time to strive in both teaching and research could be exhaustive. Some participants had administrative duties besides teaching obligations which makes it even more challenging for them to commit sufficient time and energy to research. Edward, Peter, and Zoe all assumed the dual responsibilities of teacher and administrator in their department. They felt that administrative duties had taken up much of their time and energy, which could have been spent doing research.

Finally the negative emotional experiences were also contributed by participants’ lack of research skills. The participants were well-trained in teaching as evident in their past achievements in teaching. However, in term of research, many participants raised the concern on lack of research skills including understanding theories and analyzing data. The lack of these crucial skills had hindered participants in writing and publishing, resulting in them feeling “painful,” “anxious,” and “stressed.” A review of the English department documents showed that only a handful of staff in the department have received training in research, there are only 15 teachers with a Ph.D. degree or currently pursuing one among the 83 faculty members. Among the participants, various research inadequacies caused their negative emotions. For example, Edward mentioned that his inability to conduct research at the initial stage made him feel doing research was distant and unattainable. For Betty, it was the inability to conduct empirical study, while for Mark, it was the lack of a Ph.D. degree. For Zoe and Peter, insufficient knowledge of theory also caused their negative feelings. For instance, Peter candidly stated that:

“When doing translation research now, I find it a bit difficult in trying to find a starting point for translation comparisons under the guidance of a theory. When I conduct an in-depth analysis, I feel a bit inadequate. I think my accumulation of theory knowledge is too weak.”

As for the emotions generated by the institutional system at the meso-level, participants displayed opposite emotions. While Edward, Peter, and Zoe expressed their satisfaction of the current institutional research policies, Mark, Betty, and Serena stated their dissatisfaction and powerlessness of the changes in institutional policies. According to Edward, the university’s promotion system was “very fair and just” with “a reasonable proportion of requirements in teaching, research, and social services”. Similarly, Peter showed his satisfaction when he commented on the university’s periodical evaluation system, “you can basically pass the assessment if you have certain research results within 3 years”. Zoe also showed her welcome of the corresponding changes in her university with the new research policy of breaking the “five-only” by saying:

“With the emergence of this new research policy, our school has made some timely changes. In the past, the promotion was solely based on research, right? But now it also values teaching, even though the proportion of teaching is not as high as that of research. It has made great progress in the promotion system compared with the past, so I think it is getting better and better slowly.”

However, for Mark, Betty, and Serena, the changes in the institutional policies made them feel discouraged and powerless to conduct research. According to their responses in the narrative frames and interviews, they were mainly doing research because of the “system constraint” and the “research assessment,” now they were more compelled to do so. Even though they thought that the institutional policies were unreasonable, there was nothing they could do about it, which aggravated their sense of powerlessness in doing research.

As for the country’s new research policy of breaking the “five-only,” Mark and Serena expressed negative emotion confusion about it. “Yes, you break them, but once you set up a new “four-only” or a new “three-only,” won’t that be the same thing?,” said Mark. Meanwhile, for Alice, Peter and Edward, they all asserted that it had little impact on their research even though they were in favor of it. For Alice, she was doing research at her own pace, “I’ll continue with my research when I have time”. Edward regarded doing research as part of his life, this new policy “does not have much impact”, because he “still has to continue to do research work”. Peter expressed the similar stance when he explained his attitude toward this policy:

“First of all, I am in favor of breaking, but how to build a more humane and better system than this? This requires careful consideration. The policy of breaking the “five-only” has little impact on my research, because now, I am doing research purely based on my interest, I don’t adjust my research area on that.”

### University English as a Foreign Language Teachers’ Emotion Regulation Strategies in Research

The collected data indicated that all the participants mainly regulated their emotions in research practice through antecedent-focused and response-focused approaches. Table 4 summarizes the participants’ emotion regulation approaches and specific strategies.

#### Antecedent-Focused Regulation

In the case of this study, some participants employed the strategy of situation selection to regulate their negative emotions in research. For example, Alice said that she has spent at least 10 years intermittently doing research which she felt quite fulfilling because she has constructed her researcher identity in the process. However, she also confided that her research has been stagnated for some time in recent years which caused some negative emotions for her. In order to ease the negative emotion, she completely stopped thinking about doing research for now. In her words:

“I know how to be a qualified or even excellent researcher, but I don’t have time for that now. Now I’ve been at a standstill due to family reasons, I have regrets of course, but there is nothing I can do.” “I don’t have the plan to apply for the professional title of full professor now, so I don’t think too much about that in my life.”

To modify emotional impact, some participants implemented the strategy of situation modification through changing their research practice. For instance, Peter has changed his research habit of releasing a translation book every year after he carefully studied the promotion system of his university because he discovered that translation books were not much valued in the system. Therefore, in order to avoid the possible disappointment, he promptly put an end to this research habit. He came to a conclusion that:

“We need to consider some practical issues. You can’t just keep your head down and do research in your own way. On the premise that you have enough energy to learn, you can study the terms of the promotion system because it is at least beneficial and harmless.”

Attention deployment was also used by some participants when they selected which aspect of a situation to focus on. They directed attention away from the antecedents of their negative emotions or redirect their attention based on their current focus. For example, Alice faced great pressure when she realized she had to change her research area if she wanted to proceed further. However, she still hasn’t found the new research direction due to various reasons. To ease the tension and stress, Alice redirected her attention to taking part in teaching competitions instead of focusing on locating her new research direction in the past 2 years:

“At present, it seems that I still have no new direction in my area of literature research, mainly because of family reasons, and the teaching pressure is actually quite heavy, too. So, I’ve spent a lot of time in teaching which also squeezes out my time in research. My team has been busy participating in teaching competitions for the past 2 years, so my research has been held up.”

Edward, on the other hand, redirected his attention to help himself better conduct research. He admitted that he had uncomfortable feelings such as frustration when his grant application or manuscript was rejected, at moments like that, he would do certain things to redirect his attention:

“In any case, it is definitely uncomfortable to have such a rejection, and it takes me some time to make adjustments. In the process of adjustment, I usually think about some of my successes in the past, or think about other things, try to soothe the feelings first, and then look at my manuscript or grant application again after some time.”

Another regulation strategy that has been commonly used by participants was cognitive change. Participants mainly readjusted their perspective of viewing research and research-related events in their lives, usually from a negative perspective to a neutral or even a positive one. For example, Serena, as a struggling researcher who felt powerless to change the *status quo* of great pressure in both teaching and research, mentioned that she tried to look at it from a positive perspective:

“You have to convert pressure into motivation, and you should think about the positive side.” “The promotion system of my university requires us to take both teaching and research into account, which can be regarded as a kind of motivation to our research to a certain extent.”

Similarly, Zoe, as a diligent researcher who valued the importance of research, also experienced a similar cognitive change when she constructed her researcher identity:

“I think our main identity is still a teacher, and then maybe because of the need of getting professional titles, we need to do some research work. My feeling is that it is actually worth it. I think university teachers, you can’t just go to class and teach only based on your personal experience, right? We still need some theoretical guidance.”

It can be seen that by revising the cognition of research, teachers reexamined the role of research from a positive perspective, hence, the purpose of regulating emotions can be achieved.

#### Response-Focused Regulation

Data revealed that response-focused regulation approach was used by more participants than the antecedent-focused approach. In this study, response-focused regulation included strategies of suppression, adapting, taking actions and self-improving.

When it comes to the negative emotions related to research, some participants used the strategy of suppression to weaken the influence of negative emotions. For example, Mark forced himself to be engaged in research activities by suppressing his dislike of research, “I’m not interested in research. Who would like to sit there and write manuscripts if it is not for promotion?” Another participant Serena also suppressed her emotions and continued to do research even though she thought there was some unfairness of the promotion system in her department:

“The main thing I don’t like is the unfairness in research. For example, the promotion policy in my department may change from time to time or the policy is always inclined to favor some people intentionally or unintentionally, which I think is not conducive to doing research. We teachers feel very much at a loss, but there is nothing we can do about it.”

Facing the university’s promotion system, some participants employed the strategy of adapting, that is, adjusted their feelings or mentality to regulate emotions as well as to adapt to the social-institutional context (Gu and Gu, 2019). In Mark’s words, doing research is “not something that you want to do, it is something that you have to do” since it was required by the promotion system. Likewise, once Zoe realized that she needed research achievements to apply for the professional title, she “started to turn to research” in addition to teaching. In the same way, Edward explained why and how to adapt to the promotion system:

“As for the promotion system, I think it is basically reasonable, because it has a reasonable proportion of requirements for our teaching, research, and social services.” “It is a baton for my research work. Because in the promotion system, it clearly lists which manuscripts are high-level manuscripts and which level of research grants are required, these naturally become the main focus of my research work.”

Different from Gu and Gu’s (2019) findings that teachers only take actions once they want to enhance their positive emotions, data showed that most participants in this study used the strategy of taking actions to regulate both negative and positive emotions in research. For example, Mark was a doubter of research and the new research policy; however, he still made an effort of writing manuscripts, “Just keep writing manuscripts, what else can you do?” Similarly, Peter, Serena, and Betty also took certain actions to try to be more engaged in research practice, such as “reading a lot of literature” (Peter, Serena), frequently “attending academic conferences” (Serena, Betty), and actively “applying for research grants” (Serena). Betty further explained that regularly participating in academic conferences with her team “played a driving role” for her because she needed to “bring something like abstracts to the conference.” As a firm believer in the necessity of research, Zoe also used the strategy of taking actions to promote research competence as well as augmenting positive emotions. She mentioned that she didn’t have much time to do research when she was swamped with administrative duties as head of her division:

“So, I basically got up at 4:00–4:30 every day. Whether it was winter or summer, I persisted for about 3 years. I got up early in the morning and then spent about one and a half hours reading and writing manuscripts. I think my gain is quite big.”

Data indicated that the most common strategy used by participants was self-improving. Participants expected to be a stronger version of themselves when they faced unfriendly factors in the research environment (Gu, 2016). In this study, six participants used this strategy regarding the improvement of research abilities in order to reduce the influence of negative emotions as well as enhancing positive ones. While Mark mainly “relied on self-study to improve research abilities,” Betty and Serena thought that their current pursuit of a doctorate helped them promote research competency. Zoe stated that she routinely improved herself “in a certain respect according to the requirements of that period” because “different periods have different requirements for teachers’ abilities”. Similarly, Peter habitually read all the manuscripts published in two core translation journals monthly to “keep up with the forefront of the translation discipline”. For Edward, when his grant application was not approved and made him feel frustrated, he would thoroughly reflect and revise his application form to improve its quality:

“Reflect on whether there are any shortcomings in my application form, and then try to figure out which problems can be solved by myself, and which ones can be solved by asking others for help. In this way, I will further polish my application form and continue to apply for grant next time.”

## Discussion

### Participants Experienced Wide-Ranging and Varied Emotions

This qualitative study explored the emotions that university EFL teachers experienced in research and whether and how they regulated their emotions in the context of China’s new research policy of breaking the “five-only.” Based on the research findings, it can be seen that participants all experienced wide-ranging emotions in research practice, including positive, negative and mixed emotions at micro, meso, and macro levels of research and research policy.

The participants’ positive emotions were related to their intrinsic research interests and beliefs of the mutually beneficial relationship between teaching and research. Some participants felt that research could give them enjoyment, pride and happiness with research progress and achievement, and help them improve their teaching as well. Therefore, they were affirmative of the necessity and importance of conducting research as university EFL teachers. These results echo with Bai et al. (2014) research findings that college English teachers recognize the significance of research for teaching, personal growth and professional development. Nevertheless, participants experienced positive emotions for different reasons. While some participants who were driven to do research largely based on their own interests enjoyed research and their successes in getting research grants and manuscript publications, other participants driven by external factors such as the promotion system only enjoyed research when they completed a research task. Additionally, while previous studies found that extrinsic motivation was the driving force for university EFL teachers’ research practice (Barkhuizen, 2009; Borg, 2009; Borg and Liu, 2013; Xu, 2014; Teng, 2019) or their intrinsic motivation was not strong enough to sustain their efforts to do research (Chen and Wang, 2013; Long and Huang, 2017; Xu, 2020), this study supplemented these research findings by showing that the importance of extrinsic and intrinsic motivations differed in the participants’ research engagement. For those participants who doubted the necessity of research and were struggling to get professional growth, extrinsic motivation such as getting senior professional titles and passing the periodical assessment was their main reason for research engagement. As a result, they also had more negative emotions than positive ones toward research and research policies. In contrast, intrinsic motivation such as research interests was the primary driving force for those who had firm research beliefs. They consequently had a positive or neutral attitude toward changes in institutional systems and research policies. Furthermore, a few participants were found to experience both bitterness and sweetness in conducting research. This result is similar to that reported by Gu (2016).

In line with previous research, this study also found out that participants experienced a number of negative emotions in research practice. Some university EFL teachers, as discovered in prior studies (Chen and Wang, 2013; Gu et al., 2014), were doubtful about the meaning of research to teaching as college English teachers. They didn’t think doing research could do much good to their teaching and they were driven to do it mainly by external factors such as promotion. In addition, participants in this study also showed negative emotions such as frustration and stress in doing research as reported in prior studies (Borg, 2010; Lee, 2014; Long and Huang, 2017). Other negative emotions reported in the present study included inadequacy, pain, anxiety, powerlessness, and dissatisfaction. Compared with previous studies which explored teachers’ negative emotions in research from a general picture (Xu, 2020; Wang and Han, 2011; Zhang, 2014), this study revealed the different negative emotions experienced by participants during a turbulent process of change caused by the release of China’s new research policy of breaking the “five-only.” Those who were actively engaged in research practice because of intrinsic reasons experienced negative emotions mainly stemming from doing research itself, whereas those who were driven by extrinsic motivations experienced more negative emotions which incorporated both research-related and policy-related ones. This was scarcely mentioned in previous studies because studies on research policy of breaking the “five-only” mainly discuss the repercussions of the new policy from the sociocultural and institutional levels (e.g., Cao, 2019; Luo, 2020). Taken together, the wide-ranging and varied emotions experienced by participants highlight the importance of their emotion regulation to their research practice and professional well-being.

Furthermore, data also showed that the participants had varied emotions and attitudes toward China’s new research policy and the subsequent changes in their institutional policies. Participants in this study presented three types. One embraced the changes brought about by breaking the “five-only” in the institutional policies and optimistically believed that “it was getting better and better.” She represented the type of optimistic supporter of the new research policy. Three displayed a generally indifferent attitude toward this new policy in spite of their approval of the implementation of the policy in the research policies of their institution. They claimed that the new research policy had little impact on their research practice. The reason why they were immune to the changes caused by this policy is that they adapt to various research contexts solely based on their own research beliefs and interests. They can be categorized as neutral adaptors. Unlike them, another three participants mainly displayed their negative emotions and attitudes toward the implementation of this policy in the institutional system, and complained about the negative impact on their research caused by the more demanding requirements in various aspects brought about by this policy. They stand for the pessimistic doubters of this new research policy. No matter which type the participants belonged to, they all underwent excessive stress or troublesome feelings during the unstable and turbulent process of change and policy implementation. This finding of the study calls for the attention of higher education stakeholders, who could keep track of how changes regarding research policies are being perceived and experienced by university teachers, and could carefully take the contextual reality into account in policy making and implementing so as to help teachers find it worthy of doing research in spite of contextual constraints (Clegg, 2008; Gao et al., 2011; Tran et al., 2017; Huang et al., 2018).

### Participants Used Multiple Emotion Regulation Strategies

With regard to the second research question, data indicated that participants used two approaches of emotion regulation, antecedent-focused approach and response-focused approach to regulate their emotions in research. While the former included strategies of situation selection, situation modification, attention deployment and cognitive change, the latter consisted of suppression, adapting, taking actions and self-improving, This research finding suggests that teachers, as reported in Taxer and Gross’s (2018) study, use a variety of emotion regulation strategies to adapt to sociocultural contexts. While the various strategies of antecedent-focused regulation reflect the participants’ ability to avoid negative emotions by changing their own research goals and views, the response-focused regulation strategies reflect the compromise of the participants under the great pressure of the sociocultural environment they are in.

While many strategies are associated with reducing negative emotions (Sutton and Harper, 2009; Chang, 2013; Jiang et al., 2016), this study also revealed that some strategies are related to teacher agency and aimed at enhancing positive emotions. Taking the cognitive change strategy as an instance, participants gradually agreed that research is important for their professional development, even though some of them doubted the necessity of doing research for university EFL teachers. Therefore, they reinterpreted the meaning of the research by implanting a positive interpretation of its significance into their minds, and consequently generating more positive emotions in the process. In addition, strategies of adapting, taking actions and self-improving are all the results of participants’ active choices, which reflect teacher agency and their adaptability to the situation they are in.

Similar to the findings of previous studies on Chinese teachers’ emotion regulation strategies (Gong et al., 2013; Jiang et al., 2016; Yin, 2016), suppression and adapting were among the common strategies used by participants in this study. Participants tended to suppress their negative emotions toward research and adapt to the research requirements in their institution when they could do nothing to change the *status quo* but to follow the rules. However, the study unexpectedly found that the most frequently and widely employed strategies by most participants were taking actions and self-improving, which were less discussed in prior studies. Participants used these two strategies not only to reduce the influence of negative emotions, but also to enhance their positive emotions. They took actions not only when they wanted to promote their research competency, but also when they suffered from negative emotions induced by setbacks in research practice. Similarly, they used the strategy of self-improving to enhance their positive emotions and mitigate the influence of their negative emotions in research.

Furthermore, data showed that participants used response-focused regulation approach more frequently than antecedent-focused approach. Participants mainly used this approach to regulate their emotions which were already underway in research practice and to adapt to the socio-institutional context they were in. This result was similar to that reported by Gu and Gu (2019) in their case study of university EFL teachers’ emotion regulation strategies in research. Additionally, most participants in the study were found to employ more than one regulation strategy, and four of them used both antecedent-focused and response-focused approaches to regulate emotions which reflected the multifaceted and dynamic nature of teacher emotions (Benesch, 2013, 2018; Han and Xu, 2021). Many strategies were used simultaneously and flexibly based on participants’ needs and focus at the moment so as to augment positive emotions as well as reducing the negative ones. In this way, more positive emotions could be generated and they could play a positive role in promoting teachers’ research practice, thereby improving teachers’ sense of job satisfaction and professional well-being.

### Participants’ Emotion Regulation Strategies Were Influenced by Sociocultural Context

As Mesquita and Albert (2007) pointed out that cultural differences play an important role in people’s preference of different emotion regulation strategies, the present study also found out that sociocultural context had undeniable influence on university EFL teachers’ emotion regulation strategies. Unlike teachers in Western countries who tend to use direct expressions to regulate emotions (Gu, 2016; Yin, 2016; Gu and Gu, 2019), the findings of this study indicated that Chinese teachers mostly adopt strategies to suppress their emotions, which reveals the influence of Chinese sociocultural environment on the selection of their choices. For example, some participants used the strategy of adapting to comply with the school’s research requirements whereas others used the strategy of suppression to deal with their negative emotions. These two strategies are the most common and significant manifestations of teachers’ emotion regulation in the oriental cultural environment (Jiang et al., 2016; Gu and Gu, 2019).

While Western cultures encourage people to express their positive emotions, oriental cultures such as Chinese culture highly praises people’s controlling of emotions and expressions of mild emotions (Mesquita and Albert, 2007). This was also proved in this study regarding participants’ emotions and emotion regulation strategies of China’s new research policy of breaking the “five-only.” Most participants, no matter whether they were neutral adaptors or pessimistic doubters, had negative emotions such as indifference, confusion, dissatisfaction toward this research policy and its implementation. However, they had never openly expressed their negative emotions in public. Instead, they used numerous response-focused and antecedent-focused strategies to suppress their negative emotions and to adapt to the new research requirements, which reflects the strong influence of the socio-cultural context on their choices of regulation strategies because Chinese culture greatly values individuals’ fitting in the society and maintaining harmonious interdependence with each other (King and Chen, 2019).

## Conclusion and Implications

This qualitative study explored university EFL teacher-researchers’ emotions and emotion regulation strategies in the context of the new research policy breaking the “five-only” in China. The participants were found to experience wide-ranging and varied emotions at the micro-level, meso-level, and macro-level of research, with more frequent and diverse negative emotions than positive ones. Furthermore, the participants held different attitudes toward China’s new research policy. For the optimistic supporter, she mainly experienced positive emotions and displayed her supportive attitude toward the new policy. Meanwhile, neutral adaptors showed a mixture of positive and negative emotions with a neutral attitude toward the new policy. In contrast, pessimistic doubters experienced more diverse negative emotions in research practice. Additionally, they held a pessimistic attitude toward the new policy and expressed their dissatisfaction with the changes in their institutional research policies. In order to regulate emotions and weaken the negative influence, participants used multiple emotion regulation strategies of antecedent-focused and response-focused approaches. Among the various strategies, Chinese university EFL teacher-researchers most commonly used strategies of self-improving, taking actions, adapting, suppression, and cognitive change to adapt to the socio-institutional situation they were in. The frequent use of these strategies also reflects the great influence of sociocultural context in teachers’ choices of emotion expression and regulation. The findings of this study have some implications for promoting university EFL teacher’s research practice and effective delivery of policy-making on the part of all stakeholders in higher education. Firstly, for policy makers and university administrators, it is hoped that they will be aware of the influence of various environmental factors on teachers’ emotions in research practice, and try their best to formulate policies that are conducive to promoting teachers’ research engagement and implement the policies effectively so as to build a good research atmosphere. Secondly, as far as teacher educators are concerned, they can guide teachers to form research teams and encourage them to carry out emotion regulation workshops to help master regulation strategies. In this way, teachers will have a better chance at effectively regulating their emotions once they have received such training. Thirdly, for individual teachers, they should give full play to their agency when the external environment is difficult to change. They need to master a variety of emotion regulation strategies, and adopt the most suitable ones at the right time. Apart from that, they can also seek external help such as asking for emotional support within their own research team.

The present study also has two limitations. One is that the study selected participants in a common public university in China. It is not one of the elite universities which enjoy a high reputation for research output. Future research may target EFL teacher-researchers in elite universities such as Tsinghua University and compare the possible different research emotions and regulation strategies of EFL teachers in elite universities and common ones. Another limitation is this study has only explored participants’ current emotions and attitudes toward research and China’s new research policy of breaking the “five-only”, they might experience some changes in emotions and research beliefs with the passage of time. Therefore, a longitudinal study is needed to explore the possible changes of teachers’ emotions and regulation strategies with their increasing research experiences in future research.

## Data Availability Statement

The raw data supporting the conclusions of this article will be made available by the authors upon request.

## Ethics Statement

The studies involving human participants were reviewed and approved by School of Foreign Studies, Anhui Polytechnic University. The patients/participants provided their written informed consent to participate in this study.

## Author Contributions

HL was mainly in charge of research methodology, data collection, analysis, and drafting of the manuscript. XZ was responsible for data analysis and revision of the manuscript. Both authors contributed to the article and approved the submitted version.

## Conflict of Interest

The authors declare that the research was conducted in the absence of any commercial or financial relationships that could be construed as a potential conflict of interest.

## Publisher’s Note

All claims expressed in this article are solely those of the authors and do not necessarily represent those of their affiliated organizations, or those of the publisher, the editors and the reviewers. Any product that may be evaluated in this article, or claim that may be made by its manufacturer, is not guaranteed or endorsed by the publisher.

## Appendix

Interview questions with participants:

(1) How do you see the role of teaching and research in your work?
(2) What motivates you to do research?
(3) What emotions did you have toward research before you became a researcher? What emotions do you have now?
(4) How do you regulate the emotions induced by research?
(5) What’s the most impressive event in your research life so far? How do you feel about it?
(6) What are the three things you like and dislike of doing research in your university?
(7) What do you think of the promotion system in your university? How do you feel about it?
(8) What do you think of the new research policy breaking the “five-only”? What effects does it have on your research?
